# Age-Friendly Initiatives at Arizona State University: Building Transformative Learning Systems for All

**DOI:** 10.1093/geroni/igaa057.1753

**Published:** 2020-12-16

**Authors:** Richard Knopf, Craig Talmage, Abby Baker

**Affiliations:** 1 Arizona State University, Phoenix, Arizona, United States; 2 Hobart and William Smith Colleges, Geneva, Arizona, United States

## Abstract

The AFU global initiative begin with a collaborative agreement by Arizona State University (ASU), Dublin City University (DCU), and the University of Strathclyde in 2012. The vision was to spur other universities to be fully inclusive of all generations. In 2013, ASU and DCU created a Trans-Atlantic Catalyst Fund to spur AFU initiatives at each institution, with an early focus on research in on dementia, technologies for “aging in place,” challenges of caregiving, healthy and active aging, and retirement community design. Since then, ASU has embedded Age Friendly practices throughout the institution via creation of a new foundational charter (success is “measured not by whom ASU excludes, but by whom ASU includes”), creation of ASU-wide Center for Innovation in Healthy and Resilient Aging, a re-purposed Osher Lifelong Institute, construction of the Mirabella community (twenty-story gateway for older adults into ASU), new inter-generational learning platforms, and implementation of universal learning systems.

